# Reversed shoulder arthroplasty with inversed bearing materials: 2-year clinical and radiographic results in 101 patients

**DOI:** 10.1007/s00402-014-2135-0

**Published:** 2014-12-25

**Authors:** Ulrich Irlenbusch, Max J. Kääb, George Kohut, Jerome Proust, Falk Reuther, Thierry Joudet

**Affiliations:** 1 Department of Orthopaedic Surgery, Marienstift Arnstadt, Wachsenburgallee 12, 99310 Arnstadt, Germany; 2 Sporthopaedicum Straubing, Bahnhofplatz 27, 94315 Straubing, Germany; 3 Hôpital Cantonal Fribourg, Route de Bertigny, 1708 Fribourg, Switzerland; 4 CHU de Limoges, 2 avenue Martin-Luther-King, 87042 Limoges Cedex, France; 5 DRK Kliniken Berlin Köpenick, Salvador-Allende-Strasse 2-8, 12559 Berlin, Germany; 6 Clinique du Libournais, 119 avenue de la Marne, 33500 Libourne, France

**Keywords:** Scapular notching, Complications, Reversed total shoulder arthroplasty, Inversed shoulder prosthesis, Osteolysis, Safety

## Abstract

**Introduction:**

This study documents 2-year clinical and radiographic results following reversed total shoulder arthroplasty using a novel prosthesis with inverted bearing materials (polyethylene glenoid; metal humeral component). This design was intended to avoid massive PE abrasion on the humeral side. Therefore, we predicted a lack of subsequent osteolysis-induced exacerbation of scapular notching, and because of other design features and modified operating technique a reduced notching rate.

**Materials and methods:**

An ongoing, prospective, international, multicenter study of patients implanted with a novel prosthesis at six European centers. The current analysis presents 2-year follow-up data (patients operated between December 2007 and July 2009). Clinical evaluation tools comprised the Constant–Murley score (CS), the American Shoulder and Elbow Surgeon score, range of motion, and a visual analog scale to assess pain and satisfaction. Radiographs were evaluated for notching and radiolucent lines. Any complications were recorded.

**Results:**

In total, 113 prostheses (113 patients) with a mean follow-up of 27.6 (±3.6) months were analyzed. CS increased from 22.5 (±13.7) to 65.3 (±14.9) points (*p* = 0.06). Inferior scapular notching (only grade 1 and 2) was identified in 20.5 % of patients, with no signs of PE-induced osteolysis. 4.4 % of patients experienced an implant-related complication.

**Conclusions:**

Inversion of the materials led to another type of notching with no signs of PE-induced osteolysis and no increase in the risk of short-term complications. Clinical results were comparable with other prostheses. Mid- to long-term results are required before any firm conclusions on clinical outcome and survival can be drawn.

## Introduction

Very high complication and revision rates have been reported following reversed total shoulder arthroplasty (RTSA) [1]. Scapular notching has been suggested to lead to worse clinical outcomes and potential implant failure [2]. The term refers to “erosion of bone of the scapular neck secondary to mechanical abutment of the humeral implant with adduction of the upper extremity” [3]. As the humeral implant is generally made of polyethylene (PE), this repeated abutment causes wear that in turn creates wear debris or loose particles. These may provoke a biological response leading to osteolysis [4–9]. Osteolysis may then increase the notch size by further bone degeneration.

In addition to the design and materials of the prosthesis, there are many factors influencing the incidence of scapular notching (e.g. scapular neck angle, diameter of the glenosphere, humeral inclination, surgical approach, baseplate position). This is reflected in the wide range of incidence rates for RTSAs with the same implant geometry (44–96 %) [3].

The current paper contains 2-year clinical and radiographic outcomes of an inverse shoulder prosthesis with inverted materials (PE glenosphere; metal inlay). The rationale is to avoid PE-bone contact, and thus avoid resulting PE wear and potential osteolysis. This inversion of materials appears to have no biomechanical impact [10].

We hypothesized that this implant would have in the short term: (1) clinical results and complication rates (other than scapular notching) that are comparable to implants with a similar design but without inverted materials; (2) a lack of wear-induced osteolysis, evidenced by a different radiographic appearance of the notching; (3) a reduced incidence rate of notching.

## Materials and methods

**Fig. 1:**
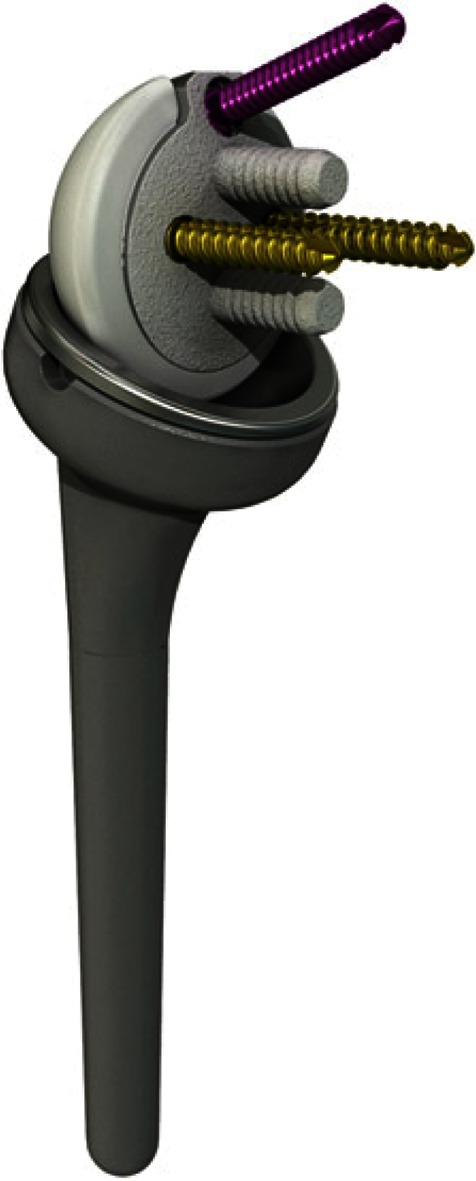
The evaluated prosthesis with a stem for cementless implantation. The metaglene is also designed for cementless implantation, and fixed with two inline pegs, one anterior and one posterior lag screw, and one superior polyaxial locking screw

This prospective, international, multicenter study enrolled consecutive patients from three sites in Germany, two in France and one in Switzerland. All patients who received an Affinis^®^ Inverse (Mathys Ltd. Bettlach, Switzerland) total shoulder prosthesis (Fig. 1) were included sequentially, except those undergoing revision of a reversed prosthesis. The study is currently on-going and will enroll around 400 patients. However, for this presentation of the initial data, only those patients who were operated on between 12 December 2007 and 25 July 2009 and had undergone a 2-year follow-up were examined. Patients were clinically and radiographically followed up at 6 weeks and 3, 6, 12 and 24 months after surgery.

Ethics committee approval was provided by the Comité Intercantonal d’Éthique (Switzerland) on 24 September 2008 (number 01/2008), and all procedures were in accordance with the Declaration of Helsinki.

### Prosthesis design

Three sizes of the PE glenosphere (36, 39 and 42 mm) and three metal inlay thicknesses (0, 3, 6 mm) for each glenosphere diameter are available for this prosthesis. The monoblock humeral stem can be anchored either with or without cement. For cases requiring revision of a non-reversed prosthesis, longer stems and revision metaglenes were available. The standard metaglene is fixed with two short parallel pegs, one superior angular stable locking screw, and two lag screws (anterior and posterior).

Design features intended to reduce mechanical notching include an eccentric metaglene (allows a more inferior position of the glenosphere), chamfering of the medial edge of the humeral inlay, and diameters of the glenosphere and humeral component that were larger than 36 mm.

The key design feature intended to reduce PE-induced osteolysis is the inversion of the glenosphere (now made of PE) and inlay material (now made of cobalt chrome).

### Operating technique

Operations were performed according to current surgical recommendations, first described by the study group of Gerber [11]. The prosthesis was inferiorly positioned to get an overhang of the lower edge of the glenoid [11, 12]. For precision, a drill guide was used by placing the inferior border of the guide precisely against the inferior rim of the glenoid.

The depth of the humeral component as well as the humeral inclination angle was not changed from the Delta III prosthesis.

### Clinical evaluation

Clinical evaluation tools included the Constant–Murley score (CS), the American Shoulder and Elbow Surgeon (ASES) score, and the range of motion (ROM) [13, 14]. ROM measured flexion/extension, abduction/adduction, internal/external rotation at 0° and internal/external rotation at 90° arm abduction. All ROM values were assessed actively and passively. Satisfaction and pain were determined using a visual analog scale (VAS). All complications were systematically recorded.

### Radiographic evaluation

All X-rays were taken according to a standard protocol followed in each center, and were evaluated for notching and radiolucent lines. The patient stood in a normal upright position and turned approximately 30° towards the involved side (true anteroposterior projection) with the arm in 30° abduction. The X-ray beams were orientated horizontally. All images were taken during expiration for minimal overlap between the prosthesis and ribcage to get an orthograde view of the metaglene and a good picture of the inferior scapular rim without being covered by the humeral component.

**Fig. 2:**
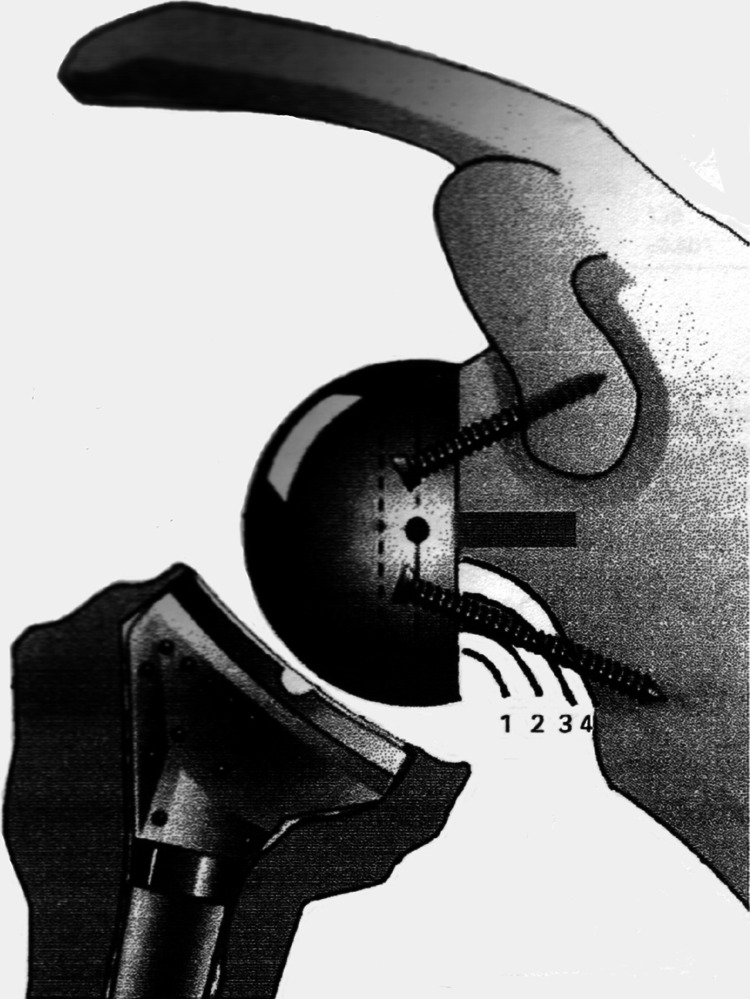
Notching classification according to Sirveaux et al. [2] and Nerot et al. [15]. Grade *1* defect confined to the pillar; grade *2* defect in contact with the lower screw; grade *3* defect over the lower screw; grade *4* defect extended under the baseplate

**Fig. 3:**
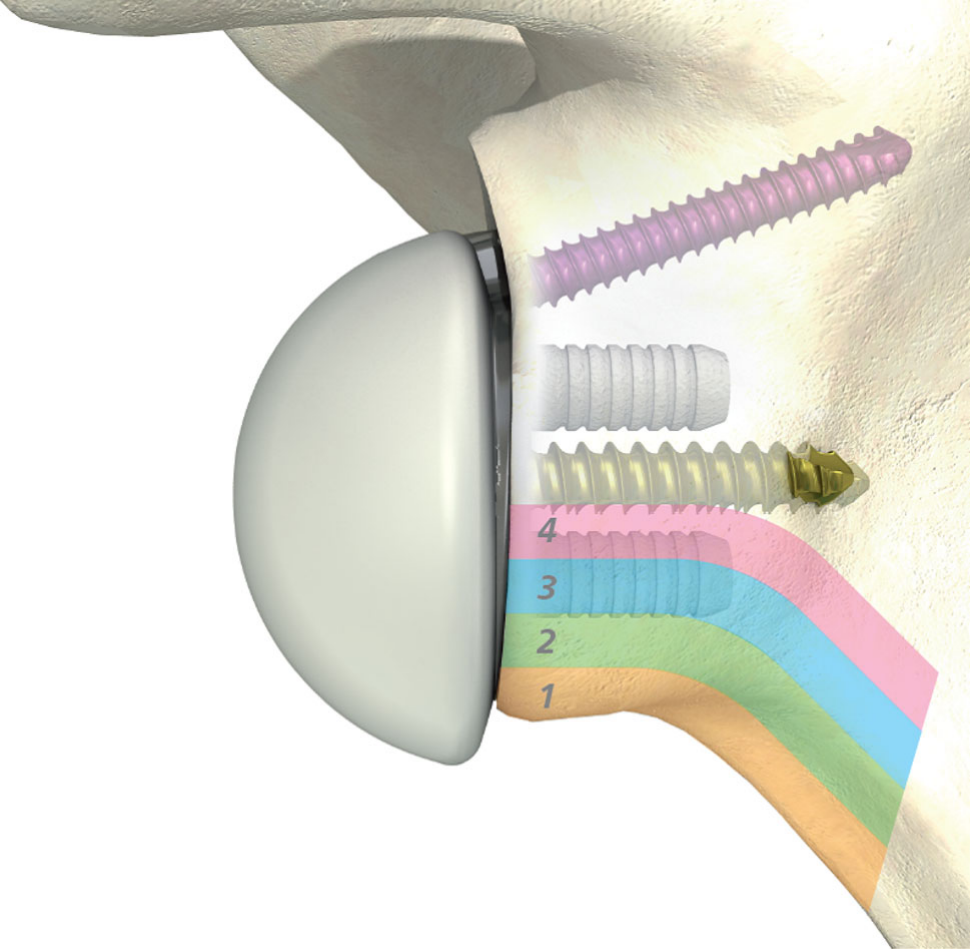
The Nerot and Sirveaux notching classification [2, 15] adapted to the evaluated prosthesis with a standard metaglene implant. Grade *1* defect extends from the inferior scapula rim to the mid-distance from the scapular rim to the inferior peg; grade *2* defect extends up to the inferior peg without peg contact; grade *3* defect extends to the middle of the inferior peg; grade *4* defect has contact with the two lag screws

As the prosthesis has no inferior screw, and the Sirveaux et al. [2] and Nerot et al. [15] classification of scapular notching uses this screw as a marker (Fig. 2), we modified the classification slightly in consultation with Prof. Sirveaux for use in this study (Fig. 3).

The degree of scapular notching visible on X-rays was first evaluated by a single author (G.K.) before being re-evaluated by the operating surgeon. Cases of disagreement were discussed between all authors until a consensus could be reached.

**Fig. 4:**
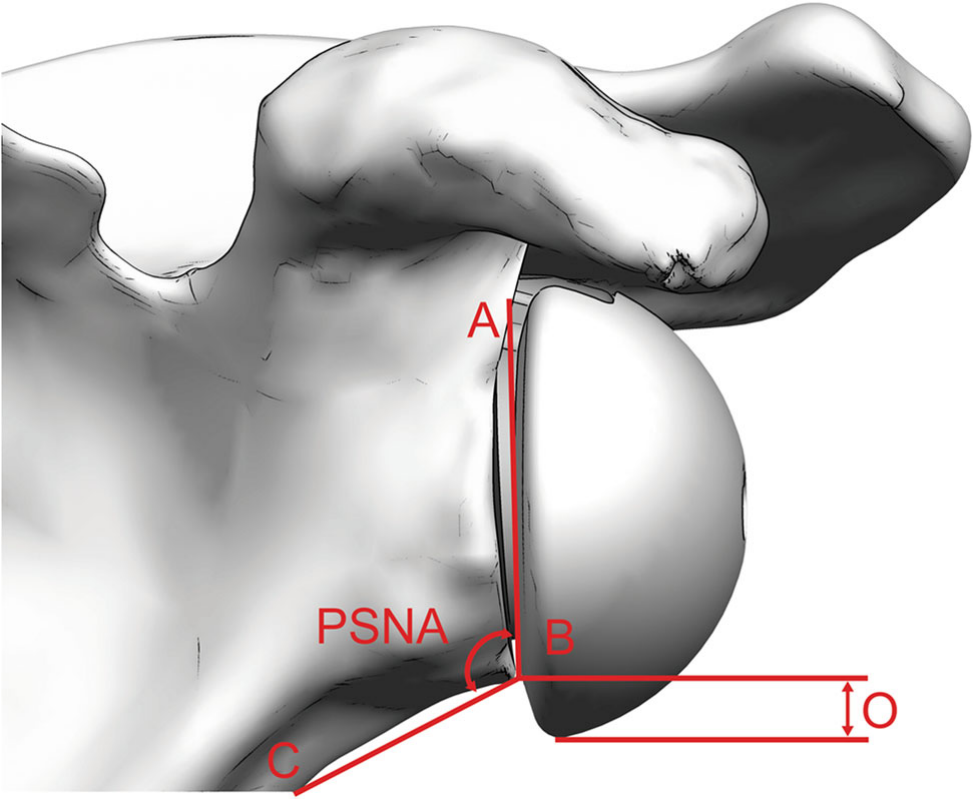
Illustration of glenosphere overhang and calculation of PSNA according to Simovitch et al. [12]. *Line AB* refers to the baseplate. PSNA is defined as the angle between *line AB* and *line BC*. Overhang (*O*) was 4.0, 5.5, or 7.0 mm depending on the size of the prosthesis used (36, 39, or 42 mm)

The overhang of the glenosphere and the prosthesis scapular neck angle (PSNA) were measured on the post-operative scapular X-ray in anteroposterior position using digital calipers and a digital goniometer (MediCAD^®^ Classic Version 2.5, Hectec GmbH, Landshut, Germany), respectively. Calibration of the X-ray measurement was done using the size of the baseplate (30.7 mm). The positioning of the baseplate in relation to the inferior rim of the scapular neck was measured, and the resulting overhang in relation to the scapular neck could be calculated (4.0, 5.5, or 7.0 mm depending on the 36, 39, 42 mm size of the prosthesis used) (Fig. 4). The PSNA measurement was done according to Simovitch et al. [12].

### Statistical analysis

Data were entered into the web-based database MEMdoc (MEM Research Center, University of Bern, Switzerland). Statistical analysis was performed using SAS software (Enterprise Guide 4.2, NC, USA).

Constant–Murley score, gender-adjusted CS, and age were tested for association with indication for RTSA using the non-parametric Kruskal–Wallis test (two-tailed). In cases of significance, a pairwise Wilcoxon test was performed. The *p* values were then adjusted according to Bonferroni for all comparisons between indications (*p* = 0.008).

To test for any systematic association between the grade of notching and indication, an exact *χ* ^2^ test of independence was performed (two-tailed). To account for the fact that the levels of parameters are ordered, a Kruskal–Wallis test was added to compare the typical grades between indications.

Influences of surgical approach and glenosphere size with presence of notching were tested using an exact two-tailed Fisher test. PSNA as well as overhang of the glenosphere with presence of notching were tested using a one-sided Wilcoxon test.

All implant-related complications were carefully recorded.

In all cases, *p* values <0.05 were considered to be significant. Data are presented as mean ± SD, unless otherwise indicated.

## Results

### Patient demographics

Between 12 December 2007 and 25 July 2009, 134 patients (137 shoulders) received the implant. Of these, 21 patients were followed up earlier than 2 years, and are therefore excluded from the current analysis, but will continue in the study. This leaves 113 patients (113 shoulders; 74.3 % females, 25.7 % males) who underwent 2-year follow-up [mean 27.6 (±3.6) months]. None of these patients received bilateral implantation. Mean age at surgery was 75.2 (±6.9) years for females and 74.9 (±7.2) years for males.

Of the 113 patients with 2-year follow-up, three patients died, eight patients could only be contacted by phone [living too far away (*n* = 1), not willing (*n* = 1), poor health (*n* = 6)], and one patient was lost to follow-up. All contacted patients indicated that the implant was in situ. This left 101 patients with a 2-year clinical evaluation and 88 patients with a 2-year radiographic evaluation.

The predominant indication was cuff tear arthroplasty (70.8 %). Less common indications were: revision from primary shoulder arthroplasty (12.4 %), fracture sequelae/posttraumatic arthritis (10.6 %), and other indications (6.2 %). Other indications included, for example, shoulder dislocation, primary osteoarthritis and primary fracture. There was a statistically significant relationship between indication and age (Table 1).

**Table 1:** Relationship between age and indication

	*n*	Age, years				
		Mean	SD	Min	Median	Max
Cuff tear arthropathy	80	76.2	5.5	54.0	76.4	87.5
Revision	12	68.4	9.4	49.8	69.5	84.8
Fracture sequelae/posttraumatic osteoarthritis	14	73.7	8.3	57.5	72.0	90.6
Other	7	77.8	8.4	69.7	74.4	93.6
Total	113	75.1	6.9	49.8	75.2	93.6

There was a statically significant relationship between age and induction (Kruskal–Wallis test two-sided, *p* = 0.0104)

*n* number of patients/prostheses, *SD* standard deviation

One-third of the patients (34.5 %) had previously been operated on the replaced shoulder. More than two-thirds of the patients (72.6 %) were operated on the right shoulder. 92.0 % of patients were right-handed, and most implantations (78.8 %) occurred on the dominant side.

The deltopectoral was used in 52 % of implantations and lateral (deltasplit) approach in the remaining 48 %. In 53 % of patients, a cemented stem was implanted; in 44 % of patients, a cementless stem was implanted; 3 % of patients received a longer cemented revision stem.

### Clinical outcome

**Fig. 5:**
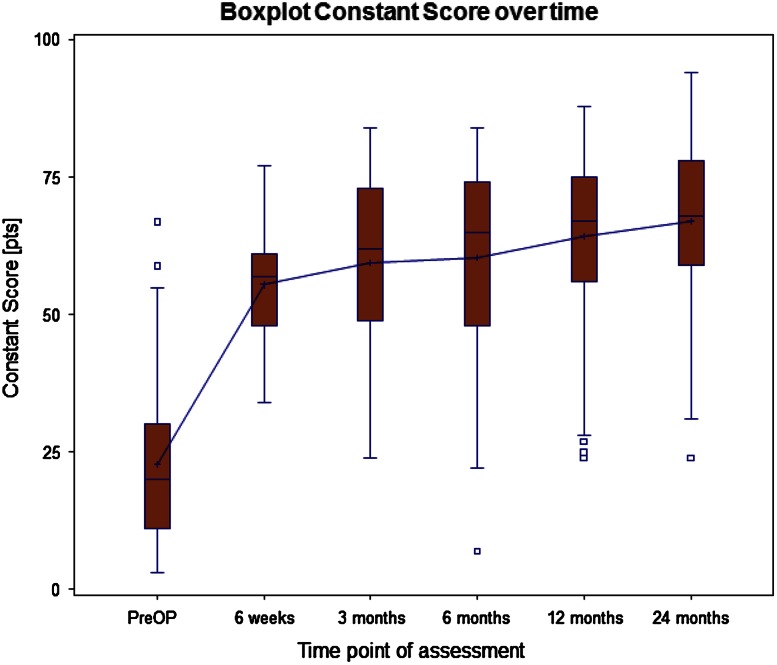
Boxplot of Constant–Murley score (CS) over follow-up time (median and mean values, interquartile range 25 and 75 %, min., max., *o* outlier that lies between 1.5 and 3 times the interquartile range)

In the 101 prostheses with 2 year clinical data, the overall CS increased from 22.5 (±13.7) to 65.3 (±14.9) points (*p* = 0.06) (Table 2; Fig. 5) and the adjusted CS from 32.4 (±19.7) % to 95.6 (±23.4) % (*p* = 0.04). Two years after surgery the CS values for pain and force improved considerably, increasing from 2.0 (±3.3) to 12.8 (±3.3) points for pain (*p* < 0.001), and from 2.6 (±2.8) to 8.0 (±4.3) points for force (*p* < 0.001).

**Table 2:** CS before operation and at 2-year follow-up

Indication	Preoperative CS^‡^			24-month CS^§^		
	*n*	Mean (points)	SD	*n*	Mean (points)	SD
Cuff tear arthropathy	79	24.7	14.0	71	67.1	14.5
Revision from primary TSA	11	17.5	12.6	12	49.5	15.0
Fracture sequelae/postraum. OA	14	16.2	11.0	12	68.8	10.3
Other	6	17.2	12.4	6	68.5	7.4
Total	110^a^	22.5	13.7	101^b^	65.3	14.9

There was no statically significant improvement between preoperative and 24-month post-operative CS (*p* = 0.0617)

*n* number of patients/prostheses, *SD* standard deviation, *TSA* total shoulder arthroplasty

CS were significantly different between indications, both preoperatively (^‡^ *p* = 0.0366, Kruskal–Wallis test two-sided) and at 24 months (^§^ *p* = 0.0045)

^a^Missing preoperative CS in three cases

^b^Missing post-operative CS in 12 cases

Constant–Murley score values of the four indication groups were significantly different, both preoperatively and at 2 years (Table 2).

The ASES score improved from 21.2 (±14.2) points preoperatively to 77.4 (±17.9) points 2 years after implantation (*p* < 0.001).

Range of motion improved from operation to follow-up: active forward flexion [from 66.8 (±40.1)° to 137.0 (±30.7)° (*p* ≤ 0.001)], abduction [from 59.8 (±34.1)° to 129.2 (±34.5)° (*p* < 0.001)], external rotation in 0° abduction [from 18.6 (±16.9)° to 26.1 (±21.6)° (*p* = 0.006)] and external rotation in 90° abduction [from 34.8 (±32.1)° to 47.9 (±27.9)° (*p* < 0.001)].

Visual analog scale value for pain improved similarly during the same time period from 7.5 (±2.1) preoperatively to 1.2 (±1.9) (*p* < 0.001), and the VAS satisfaction score from 1.5 (±1.6) to 8.7 (±1.9) (*p* < 0.001).

### Radiographic findings

Of the 88 implants with 2-year radiographic follow-up, 10 radiographs could not be evaluated concerning notching due to poor quality. The mean follow-up time of the remaining 78 X-rays was 27 (±3) months. The degree of scapular notching visible on X-rays was first evaluated by a single author (G.K.). Next, the notching was re-evaluated by the operating surgeon, who was blinded to the initial score. Cases of disagreement were to be discussed until a consensus could be reached between all authors; however, this was not necessary as all ratings matched.

No notching was found in 79.5 % of the radiographs. Of those with notching, 14 cases were grade 1 and 2 cases were grade 2 (Table 3). There was no relationship between indication and grade of notching (*p* = 0.78).

**Table 3:** Notching rate at 2-year follow-up

Notching	Frequency	Percent	Percent total
Grade 0	62	79.5	79.5
Grade 1	14	17.9	20.5
Grade 2	2	2.6	
Grade 3	0	0.0	0.0
Grade 4	0	0.0	
Total	78	100.0	100.0

Total notching rate is 20.5 %. There is no significant relation between the grade of notching and indication (*p* = 0.78)

**Fig. 6:**
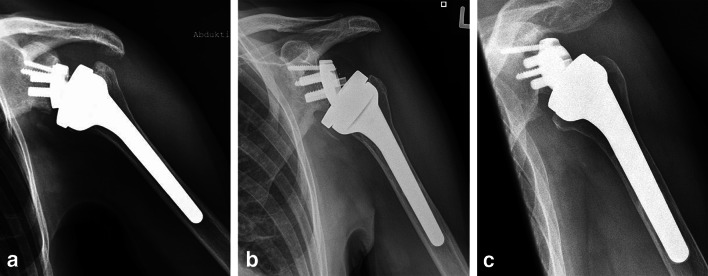
X-ray of the evaluated prosthesis: **a** initial notching (grade 1) on the inferior rim of the scapular neck. **b** Grade 1 notching. **c** Grade 2 notching. Note that the shape of the notch matches the shape of the humeral inlay, and the bone defect is located away from the metaglene without any baseplate contact

Typically, notching caused by the metal humeral implant had a different radiographical appearance than that caused by PE humeral implants: it was located away from the baseplate without any baseplate contact and reflected the shape of the humeral inlay with a sharp borderline (Fig. 6). Neither loosening nor progressive lucent lines behind the baseplate were observed in any of the radiographs. Slight differences but no significant relationship between notching and glenosphere size (Table 4) or surgical approach (Table 5) were found.

**Table 4:** Notching rate according to glenosphere size

Glenosphere size	No notching observed	Grade 1	Grade 2	Notching rate (%)
36 mm	22	7	0	24.1
39 mm	33	6	2	19.5
42 mm	7	1	0	12.5
Total	62	14	2	20.5

No significant relationship between the rate of notching and the glenosphere size was found (*p* = 0.8)

**Table 5:** Notching according to surgical approach

Surgical approach	No notching observed	Notching observed	Total
Deltopectoral	31 (79.5 %)	8 (20.5 %)	39
Lateral (Deltasplit)	31 (79.5 %)	8 (20.5 %)	39
Total	62 (79.5 %)	16 (20.5 %)	78

No significant relationship between the rate of notching and the surgical approach was found (*p* = 1)

In total 81 radiographs were available to determine the post-operative PSNA angle. The post-operative PSNA was 101 (±15)°.

Observed notching regarding glenosphere position (overhang of the glenosphere and PSNA) are presented in Table 6. Glenosphere position could be determined in 81 radiographs. PSNA was significantly different for patients with (107°, range 80–127) and without (99°, range 69–130) observed notching (*p* = 0.044). Glenosphere position was significantly different for the applied surgical approach. Glenosphere overhang was 2.9 (±1.7) mm for the deltasplit approach and 4.5 (±2.0) mm for the deltopectoral approach (*p* = 0.0001).

**Table 6:** Notching rate according to PSNA and glenosphere overhang (*n* = 81)

	No notching observed	Notching observed	Total	*p* value
PSNA (°)	99 (69; 130)	107 (80; 127)	101 (69; 130)	0.044
Glenosphere overhang (mm)	3.7 (0; 8.9)	3.7 (0; 6.4)	3.7 (0; 8.9)	0.352

Data are presented as mean (min; max). A one-sided Wilcoxon test was applied

### Complications

During follow-up, 5 (4.4 %) implant-related complications were reported. Three cases (2.7 %) sustained a shoulder dislocation, all of which were treated successfully with an open reposition and inlay elevation. Two dislocations occurred during hospital stay (1–10 days postoperatively), and one after 6 weeks. Furthermore, there was one (0.9 %) traumatic avulsion of the metaglene after a fall onto the elbow which had to be revised to a hemi-prosthesis. One case (0.9 %) had a periprosthetic fracture around the shaft which was treated with osteosynthesis (open reduction internal fixation). There was no revision for aseptic loosening or notching. To date, none of the patients have needed a revision of any of the two components.

## Discussion

This study aimed to investigate 2-year clinical and radiographic outcomes of the evaluated prosthesis with special emphasis on whether the design changes had reduced the extent and rate of scapular notching.

Our first hypothesis was that clinical outcomes and complication rates would be comparable to those of other similar implants, such as the Delta III reverse prosthesis [16, 17]. This was indeed the case, and the initial short-term clinical results look promising. Our results indicate that, at least on short-term follow-up, the inversion of bearing materials in this prosthesis appears to have no impact upon rates of complications other than scapular notching.

Our second hypothesis was that a lack of wear-induced osteolysis would result in notching with a different radiographic appearance. Indeed, the shape, borderline, size and location of notching differed from notching seen in conventional reversed shoulder designs. Scapular bone defects corresponded to the shape of the humeral inlay, were located away from the baseplate, were of smaller size, and had a sharp borderline. These findings indicate a lack of visible osteolysis. However, without histological analysis, we cannot exclude the presence of osteolysis induced by wear or, perhaps more importantly, by abrasion [10]. The authors are aware of one post-mortem study on a retrieved Delta III prosthesis that reported notching, bone loss and a chronic foreign-body reaction in the joint capsule [4]. While histological results from our study would be interesting, the results of Kepler et al. [18] suggest that they might not be informative regarding early phases of osteolysis that are not yet visible radiographically.

Our third hypothesis was that the changes to the implant design and the modified operating technique would result in a relatively low incidence rate of scapular notching. In RTSA, radiographic evidence of notching generally appears between 1.5 and 14 months postoperatively, and has a reported incidence of 44–96 % of cases [3]. The rate we found (20.5 %) was low in comparison to rates reported for similar prostheses (e.g. Delta III) over similar follow-up times (56–96 % [17, 19]) and for other prostheses that also have a medialized center of rotation (weighted mean of 63 % over 46-month mean follow-up) [20]. It should be noted that the rate we are reporting is still high in comparison to prostheses with a more lateralized center of rotation [21, 22]. However, lateralized prostheses are also associated with design-specific complications [23–27]. The choice of using a medialized or lateralized prosthesis should be made by the well-informed surgeon, and is beyond the scope of this discussion.

Without a control group implanted with a non-inverted version of this prosthesis, it is difficult to isolate the impact of inverting the bearing materials upon the severity of notching. Boileau et al. [5] have suggested that notching of grade 3 or 4 cannot be due to mechanical impingement, and is instead due to osteolysis caused by wear debris (as indicated by Nyffeler et al. [4]). If this is correct, then it would lend support to our hypothesis that inverting the bearing materials reduced wear-induced osteolysis, as we found no notching of grade 3 or 4. However, the severity of notching is influenced by factors other than implant design. For example, with the Delta III prosthesis, Simovitch et al. [12] found only 2.6 % of shoulders had notching above grade 2 (24 months), while Werner et al. [28] found grade 3 or 4 notching in 46 % of shoulders (38 months). Thus, we cannot draw any conclusions on this point.

The results presented in this paper are from a short-term follow-up. It will be of great interest to see whether the rate and extent of notching change over time. Numerous studies have reported that both increasing size and incidence of scapular notching occurs after longer-term follow-up [6, 8, 26]. However, in these studies, notch progression was inconsistent; some notches were stable after 1 year and others displayed progression even after 3 or 4 years.

In addition to lateralization or medialization of the center of rotation, there are numerous other potential influences upon scapular notching, which include—but are not limited to––glenosphere size, PSNA, and inferior glenoid position.

Several authors have reported a relationship between notching rate and glenosphere size [9, 27, 29, 30]. We did not find a statistically significant relationship, but were limited by low patient numbers in particular subgroups (i.e. only 7 patients in the 42 mm group).

Nyffeler et al. [11] showed that placing the glenosphere beyond the inferior glenoid rim significantly improved adduction and abduction angles. Roche et al. [20] showed that female patients without notching (though not male patients) showed significantly more glenosphere overhang than patients with notching. Other studies have shown no correlation between scapular notching and glenosphere positioning [31, 32]. We also found no relationship between glenosphere overhang and the presence of notching. However, we included all indications into our analysis, and subsequent evaluations of the data found that the overhang significantly differs between indications (*p* = 0.031). Therefore, the analysis will be repeated for patients with the specific indication ‘rotator cuff tear arthropathy’ once sufficient numbers have reached 2-year follow-up.

In this study, the mean PSNA of patients without notching was significantly lower than for those patients with notching. These findings support both the geometric computer analysis of Roche et al. [20] and the in vivo findings of Simovitch et al. [12].

A final point of interest concerns the replacement of the inferior screw with a peg in this implant design. In 2006, Clavert et al. [33] proposed that scapular notching is a result of micro movements in the lower screw. Recently, Day et al. [34] reported that in seven specimens retrieved at revision, rim wear was more extensive when the inferior screw had made contact with the liner. Based on these results and similar observations made in numerous other studies, it was decided to omit the inferior screw. Neither radiographic nor clinical disadvantages related to leaving out this screw have been observed.

When interpreting our data, there are some limitations to the study that should be kept in mind. Most importantly, the lack of a control group means that the data had to be compared to historical controls and literature data. Such comparisons are fraught with difficulty due to between-center differences in surgical techniques and approach, and the speed of innovation both in prosthesis design and surgery. In addition, the short-term follow-up means that our results are not yet able to yield insight into mid- and long-term survival of this prosthesis. The relationship between scapular notching and survival for this prosthesis will be known only when longer-term data become available. Another limitation was using radiographs rather than fluoroscopy, as some radiographs could not be analyzed for notching. Anterior and posterior notching was not assessed.

Long-term follow-up of these patients will provide more robust data on the clinical outcomes and survival of this prosthesis. These data may also help to distinguish the roles played by mechanical abutment and osteolysis in the degree of scapular notching.

Inversion of the materials in this new prosthesis has not led to an increased risk of complications in the short term. This inversion is the likely explanation for the failure to observe notching compounded by PE-induced osteolysis in this patient group. Mid-to long-term results are required before any firm conclusions on safety and efficacy may be drawn.
